# Anesthetic propofol epigenetically regulates breast cancer trastuzumab resistance through IL-6/miR-149-5p axis

**DOI:** 10.1038/s41598-020-65649-y

**Published:** 2020-06-01

**Authors:** Dan Tian, Miao Tian, Zhi-ming Ma, Lei-lei Zhang, Yun-feng Cui, Jin-long Li

**Affiliations:** 1 Department of Anesthesiology, The Second Hospital of Jilin University, 218 Ziqiang Street, Changchun, 130041 China; 2 Department of Gynecology, The Second Hospital of Jilin University, 218 Ziqiang Street, Changchun, 130041 China; 3 Department of Gastrointestinal Surgery, The Second Hospital of Jilin University, 218 Ziqiang Street, Changchun, 130041 China

**Keywords:** Breast cancer, Cancer microenvironment

## Abstract

Propofol, a common intravenous anesthetic, has been found to exert anti-cancer effects with inhibition of cancer cell proliferation, migration and invasion. We tested its possible action against HER2-overexpressing breast cancer cells that developed resistance against trastuzumab. Cell viability assay, ELISA for cytokines, mammosphere formation, quantitative RT-PCR for EMT/IL-6-targeting miRNAs and the *in vivo* experimental pulmonary metastasis model were performed to understand the epigenetic action of propofol. Propofol sensitized HER2 overexpressing cells to trastuzumab but such action was even more pronounced in resistant cells. Increased cytokines IL-6 as well as IL-8 were released by resistant cells, along with increased mammospheres and induction of EMT, all of which was inhibited by propofol. IL-6 targeting tumor suppressor miR-149-5p was found to be the novel miRNA that was up-regulated by propofol, resulting in the observed effects on cell viability, IL-6 production, mammospheres generation as well as EMT induction. Further, antagonizing miR-149-5p attenuated the propofol effects confirming the epigenetic activity of propofol through miR-149-5p regulation. Finally, *in vivo* validation in an experimental metastasis model conformed an inhibitory action of propofol against experimental lung metastasis and the essential mechanistic role of miR-149-5p/IL-6 loop. These results present a novel role of general anesthetic propofol against resistant breast cancer cells and the underlying epigenetic regulation of a tumor suppressor miRNA.

## Introduction

Propofol is a commonly used general anesthetic^1^. It is used for induction as well as maintenance of anesthesia. It is short acting and slows down the activity of nervous system and brain. Lately, there has been an interest in the anticancer potential of propofol^2^. Retrospectively, it has been suggested that the choice of anesthetic during oncological surgery can influence patient outcome^3^. Even though the clinical studies have provided contrary conclusions, with some supporting benefit while others finding no survival benefit associated with the use of propofol as the anesthetic of choice during cancer surgeries^4–7^, the *in vitro* data supporting a role of propofol against proliferation, invasion etc. of cancer cells is overwhelming to ignore^8,9^. Against breast cancer cells, propofol is particularly effective with demonstrated role in preventing proliferation^10^, inducing apoptosis^11^ and reducing metastasis^12^.

While there is data on anti-cancer properties of propofol in general, there is not much information on the role of propofol against the acquired resistance against therapy. Therefore, we planned this study to evaluate the ability of propofol to reverse the acquired trastuzumab resistance (Tr-R) in HER2-overexpressing cells. HER2 overexpression is known to associate with increased proliferation^13^ and metastases^14^ and given the reports on propofol against proliferation and metastasis, we thought this was an interesting topic to investigate. For our model system, we chose the HER2 overexpressing SKBR3 breast cancer cells, exposed them to trastuzumab for prolonged time to generate Tr-R SKBR3 cells and then studied the action of propofol against these cells, along with mechanistic insights. Our results showed increased production of IL-6 by Tr-R cells, which was inhibited effectively by propofol. We focused on the epigenetic mechanism of propofol action because of the recent reports highlighting such activity of propofol^15^. In addition to cell line-based studies, we also confirmed the mechanism *in vivo* in an experimental pulmonary metastasis model.

## Materials and Methods

Herceptin was obtained from our hospital’s pharmacy and diluted in bacteriostatic water containing 1.1% benzyl alcohol. Propofol was purchased from Sigma Chemical Company (China) and diluted in DMSO (vehicle) as needed. MiRNA hairpin inhibitor-miR-149-5p, or the non-specific scrambled controls were purchased from Thermo Scientific (China) and transfected using siPORT™ NeoFX™ Transfection Agent (Thermo Scientific, China).

### Cell culture

SKBR3 and HTB-20 cells, purchased from ATCC, are HER2 overexpressing breast cancer cells used in current study. These cells were cultured in DMEM media (ThermoFisher, China), supplemented with 10% FBS, in a 5% CO_2_ controlled atmosphere, at 37 °C. Cells were passaged twice a week once they reached 60–80% confluency.

### Cell counting kit-8 (CCK8) assay

Cell viability was studied by using cell counting kit-8 reagent, as per the provided instructions. 5000 cells were seeded overnight in a 96-well plate and treated with trastuzumab as indicated for 96 hrs (4 days). At the end of incubation period, CCK8 solution was added and incubated at 37 °C, followed by O.D. reading at 450 nm on a Shimadzu spectrophotometer.

### ELISA assay

IL-6 and IL-8 were measured in cell culture supernatants by ELISA, using the kits purchased from R&D Systems (China). The 4.5 h solid phase ELISA assay was performed exactly as described by the manufacturer. 100 μl supernatant was taken and added to 100 μl of assay mixture, followed by incubation for 2 h at room temperature. After 4 washes, 200 μl of conjugate was added to each sample and incubated further for 2 h at room temperature. After 4 further washes, 200 μl of substrate solution was added followed by addition of 50 μl of stop solution. Readings were taken at 450 nm on a Shimadzu instrument and wavelength correction was set to 570 nm.

### RNA extraction and quantitative RT-PCR

Total cellular RNA was extracted from and cells, using TRI reagent (Sigma Chemical Company, China). RNA was reverse-transcribed using the cDNA Synthesis Kit (ThermoFisher, China). Quantitative real-time PCR was performed with an ABI StepOnePlus™ real-time PCR System (Applied Biosystems, China) using the SYBR Green mix (ThermoFisher, China). The relative gene expression was calculated using the 2^−△△Ct^ method.

### Mammospheres culture

Cells were plated in single cell suspensions on ultra-low attachment plates (Corning, China) at a density of 1000 viable cells/ml. They were cultured in mammosphere culture medium, consisting of serum-free DMEM-F12 (ThermoFisher, China), supplemented with B27 (1:50, ThermoFisher, China), 10 ng/mL EGF (BD Biosciences, China), 20 ng/mL bFGF (Sigma, China), 0.4% bovine serum albumin (Sigma, China), 4 mg/mL insulin (Sigma, China) and heparin (Sigma, China). Cultures were allowed to proceed for 3 weeks and mammospheres calculated using a bright field microscope.

### *In vivo* mice study

We used female athymic mice (4–5 weeks old) for our study. Mice were housed under pathogen free conditions with a 12 h light/12 h dark schedule, fed autoclaved standard chow and water *ad libitum*. The study protocol was approved by the Jilin University Bioethical and Experimental Animal Care Committee, and all research was performed in accordance with relevant guidelines/regulations. 5 × 10^5^ cells were injected intravenously via the tail vein and mice were euthanized exactly after 10 weeks. Lungs were examined for metastatic nodules and metastatic nodules were removed, weighed and equal amounts (by weight, 50 mg each) were digested into single cell suspensions in SKBR3 cells’ normal culture medium. The supernatant was collected and IL-6 measured by ELISA, as described above.

### Statistical analysis

We used SPSS19.0 software for statistical analyses. Two-tailed Student’s t-test or one-way analysis of variance (ANOVA) was used to evaluate statistical difference between groups. *p* values <  0.05 were considered to be significant.

## Results

### Propofol affects trastuzumab sensitivity

**Figure 1:**
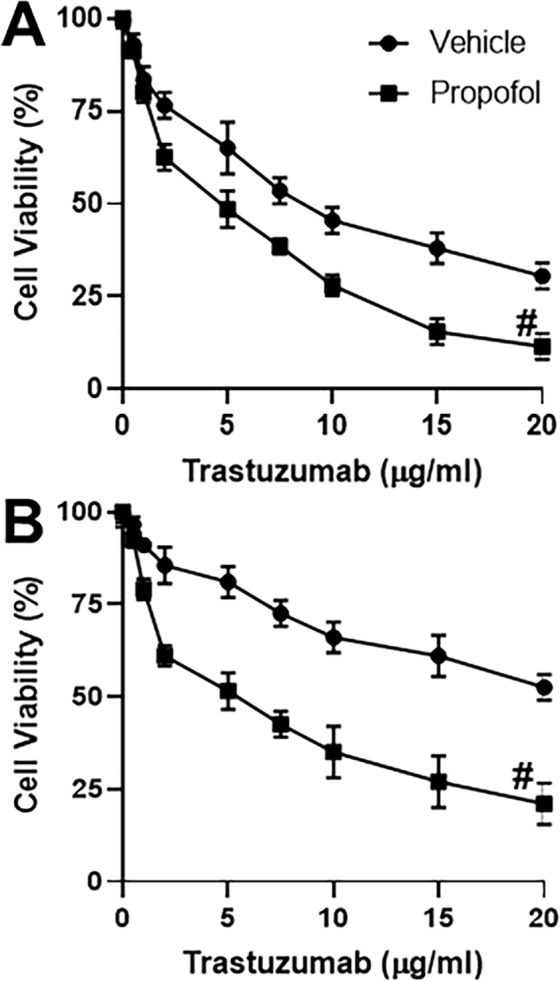
Propofol affects response to trastuzumab. Parental (**A**) or Trastuzumab-resistant (Tr-R) (**B**) SKBR3 cells were treated with increasing doses of trastuzumab as indicated on X-axis in the presence of either DMSO vehicle or 5 μg/ml propofol for 96 h. Cell viability was calculated by CCK8 assay. The number of viable cells in 0 μg/ml trastuzumab treated vehicle group were regarded as 100% and the ‘relative’ percentage in other groups was calculated. ^#^p < 0.01, compared to vehicle.

With the goal to check the effect of propofol on trastuzumab resistance of HER2 overexpressing breast cancer cells, we subjected the HER2 overexpressing SKBR3 cells to increasing doses of trastuzumab in the presence and absence of propofol. We observed that propofol increased the sensitivity of cells to trastuzumab as more cells were killed by trastuzumab in the presence of propofol (Fig. 1A). We selected the dose of propofol to be 5 μg/ml based on the published literature as well as our preliminary experiments with normal breast epithelial cells wherein this dose was non-toxic for as many as 120 hours (results not shown). Whereas the IC-50 of trastuzumab against control SKBR3 cells was 8.7 ± 0.6 μg/ml, it was decreased by propofol to be 4.6 ± 0.4 μg/ml (Table 1).

**Table 1:** Trastuzumab IC-50 values of Parental and Trastuzumab-resistant SKBR3 cells in the presence and absence of Propofol.

Condition	IC-50
Parental SKBR3	8.7 ± 0.6
Parental SKBR3 + Propofol	4.6 ± 0.4
Tr-R SKBR3	>20
Tr-R SKBR3 + Propofol	5.1 ± 0.4

Experiment described in Fig. 1 was used to calculate all the IC-50 values. Treatment was for a total of 96 hours. Propofol concentration was 5 μg/ml. Control cells (without propofol) were treated with equal volume of DMSO vehicle. All IC-50 values are in the units of μg/ml. Tr-R SKBR3: Trastuzumab resistant SKBR3 cells. >20: IC-50 could not be achieved at the highest concentration of 20 μg/ml Trastuzumab tested.

We then treated SKBR3 cells with escalating doses of trastuzumab for 3 months to generate trastuzumab-resistant (Tr-R) cells and repeated the same experiment as above. First, we noted increased resistance of these cells to trastuzumab (Fig. 1B). Moreover, we found that these cells were also sensitized by propofol to trastuzumab. The IC-50 values provided in Table 1 further support the observations. Whereas IC-50 was not achieved in Tr-R cells at the highest dose tested (20 μg/ml), propofol significantly brought it down to 5.1 ± 0.4 μg/ml.

### Propofol reduces trastuzumab resistance-associated increase in cytokine

**Figure 2:**
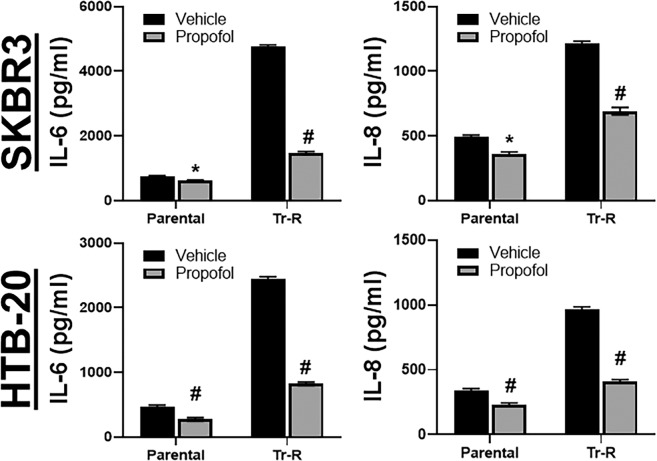
Propofol affects cytokine production. Parental or Trastuzumab-resistant (Tr-R) SKBR3 cells or HTB-20 cells were treated with either DMSO vehicle or 5 μg/ml propofol for 96 h. Cell culture supernatant was collected and ELISA was performed to quantitate levels of IL-6 or IL-8. *p < 0.05 and ^#^p < 0.01, compared to vehicle.

Since propofol inhibits the release of cytokines IL-6 and IL-8^16^ and these cytokines play a role in resistance to trastuzumab^17^, we checked for the release of these cytokines by HER2 overexpressing cells. As seen in Fig. 2, both of these cytokines were released in significantly large quantities by Tr-R cells, as compared to the parental HER2 overexpressing SKBR3 cells. Further, propofol significantly (p < 0.05) decreased their release in parental cells, but even more (p < 0.01) in the Tr-R cells. To rule-out any potential cell line-specific effects, we tested another cell line (HTB-20) as well. Similar to SKBR3 cells, HTB-20 cells were also treated with escalating doses of trastuzumab for 3 months to generate trastuzumab-resistant (Tr-R) cells. We observed similar trends as SKBR3 cells as propofol significantly inhibited the release of cytokines even though the basal levels were lower compared to SKBR3 cells. In HTB-20 cells as well, propofol inhibited cytokine release from resistant cells more than the parental cells (Fig. 2).

### Mechanism of propofol action

**Figure 3:**
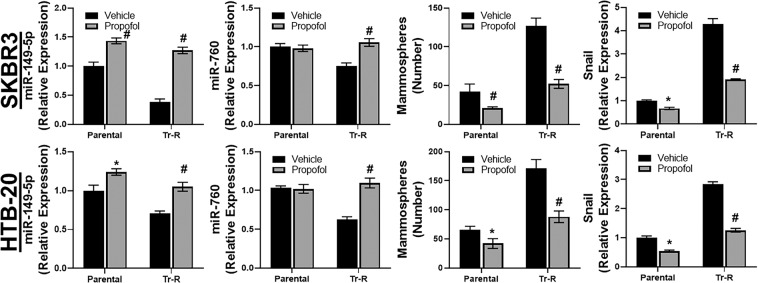
Propofol affects miR-149-5p, miR-760, mammospheres and EMT. Parental or Trastuzumab-resistant (Tr-R) SKBR3 or HTB-20 cells were treated with either DMSO vehicle or 5 μg/ml propofol for 96 h. Total mRNA was then collected from the cells and miR-149-5p/miR-760 quantitated by quantitative RT-PCR. U6 was used as an internal control for miRNA expression. Mammospheres generated, as described in Methods, were counted under different experimental conditions and levels of EMT marker, snail, quantitated by quantitative RT-PCR. For mRNAs’ internal control and normalization, we used gene expression of glyceraldehyde-3-phosphate dehydrogenase (GAPDH). *p < 0.05 and ^#^p < 0.01, compared to vehicle.

In view of the significant release of IL-6 by Tr-R cells as shown in preceding results, we focused on epigenetic basis of such release. Of note, trastuzumab resistance is controlled by epigenetic mechanisms^18^ and, moreover, such epigenetic action of propofol has been reported before^15^. We turned to Targetscan (www.targetscan.org) to list the miRNAs that can regulate IL-6, and evaluated the top two such miRNAs – miR-149-5p and miR-760. The results obtained with both of these miRNAs are presented in Fig. 3. Since these miRNAs target IL-6, they are tumor suppressors and therefore down-regulated in Tr-R cells, compared to control cells. Propofol significantly up-regulated miR-149-5p in both parental as well as Tr-R cells, with the effect being more significant in the resistant cells (p < 0.01). A similar trend was observed for miR-760 as well, however, only the effect in resistant SKBR3 as well as HTB-20 cells, and not the parental cells, was found to be significant for this miRNA (Fig. 3).

Both cancer stem cells and the phenomenon of epithelial-to-mesenchymal transition (EMT) influence therapy resistance. Therefore, we next checked for these in our cell models. SKBR3 and HTB-20 cells were grown to generate mammospheres and we found that more mammospheres were formed by Tr-R cells, relative to parental cells (Fig. 3), supporting a role of cancer stem cells in trastuzumab resistance. Propofol reduced mammospheres formed by parental cells as well as the Tr-R cells. As an EMT marker, we checked for the expression of snail and found it elevated in Tr-R cells, relative to parental cells. Similar to the effect of propofol on mammospheres, it reduced the level of EMT marker snail in parental cells, but even more in the Tr-R cells (Fig. 3).

### Inhibition of miR-149-5p attenuates propofol effects

**Figure 4:**
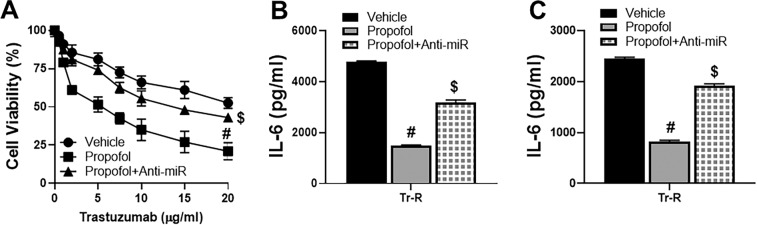
Inhibiting miR-149-5p attenuates propofol effects. (**A**) Trastuzumab-resistant SKBR3 cells were treated with increasing doses of trastuzumab as indicated on X-axis in the presence of either DMSO vehicle or 5 μg/ml propofol for 96 h. Additionally, Trastuzumab-resistant cells transfected with anti-miR-149-5p oligos (Anti-miR) were also evaluated. Cell viability was calculated by CCK8 assay. The number of viable cells in 0 μg/ml trastuzumab treated vehicle group were regarded as 100% and the ‘relative’ percentage in other groups was calculated. Trastuzumab-resistant (Tr-R) (**B**) SKBR3 or (**C**) HTB-20 cells were treated with either DMSO vehicle or 5 μg/ml propofol for 96 h. Additionally, Trastuzumab-resistant cells transfected with anti-miR-149-5p oligos (Anti-miR) were also evaluated. Cell culture supernatant was collected and ELISA was performed to quantitate levels of IL-6. ^#^p < 0.01, compared to vehicle and ^$^p < 0.01, compared to propofol alone.

With the observed up-regulation of miR-149-5p by propofol, which could explain the inhibitory action of propofol on IL-6 production, we next checked if countering such up-regulation of miRNA could attenuate propofol effects. We tested this by transfecting anti-miR-149-5p in Tr-R SKBR3 cells and subjecting them to cell viability assay. We observed that the sensitization of Tr-R cells to trastuzumab by propofol was significantly attenuated by the anti-miR-149-5p (p < 0.01) (Fig. 4A). This also resulted in similar effect on IL-6 release by the Tr-R SKBR3 cells (Fig. 4B) and Tr-R HTB-20 cells (Fig. 4C).

### *In vivo* validation

**Figure 5:**
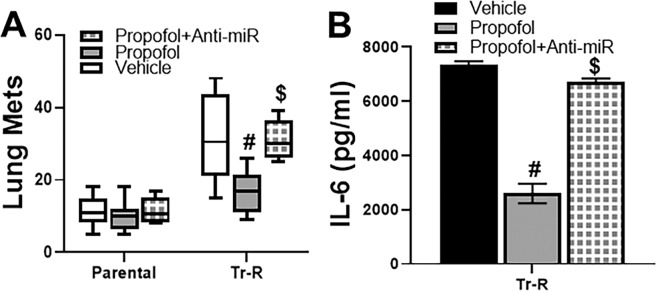
*In vivo* study. (**A**) Parental or trastuzumab-resistant (Tr-R) SKBR3 cells were injected intra-venously in athymic mice, as described in Methods. The propofol treated mice were in two different groups – either injected with cells that were transfected with non-scrambled control (propofol) or injected with cells transfected with anti-miR-149-5p oligos (propofol + Anti-miR). Lung metastatic nodules (Lung Mets) were counted in all animals (n = 8 each in each group). (**B**) The extracted modules were weighed and exactly equal amounts (50 mg) were subjected to RNA isolation and subsequent culture in culture dishes. Cell culture supernatant was collected and ELISA was performed to quantitate levels of IL-6. ^#^p < 0.01, compared to vehicle and ^$^p < 0.01, compared to propofol alone.

Finally, we took our studies to an *in vivo* model to validate the *in vitro* findings. For this, we turned to an experimental metastasis assay. Parental as well as Tr-R cells (with or without anti-miR-149-5p transfections) were injected into mice and the resulting lung metastases counted. While an effect of propofol on lung mets in parental cells was observed (Fig. 5A), the effect was much more pronounced in the Tr-R cells (p < 0.01) which, to start with, had much more metastases than the parental cells. Transfections with anti-miR-149-5p significantly (p < 0.01) attenuated the ability of propofol to inhibit lung mets. When these lung metastatic nodules were further cultured, the release of IL-6 in the culture supernatant was found to be inhibited by propofol, as expected, only to be attenuated by anti-miR-149-5p (Fig. 5B), this validating the *in vitro* findings.

## Discussion

Breast cancer is the most common cancer affecting Chinese women^19^. The incidence rate for breast cancer has increased over the last decade without the corresponding increase in mortality because of early diagnosis^20^. However, the number of women succumbing to this disease is still high, thus calling for further research. Among the many breast cancer subtypes, the HER2 overexpressing breast cancers are very aggressive with increased chances of metastatic disease^14^. Trastuzumab is used to treat HER2 overexpressing breast cancers but acquired resistance against trastuzumab is a clinical reality^18^. In the present study, we generated trastuzumab resistant cells by continuous and prolonged exposure of HER2 overexpressing cells to trastuzumab, and then evaluating the cytokine expression and the underlying epigenetic regulation.

We observed increased release of IL-6 by Tr-R cells. This is in agreement with the basic conclusion of an earlier report where an antibody against IL-6 was shown to sensitize trastuzumab-resistant tumors^21^. Thus, IL-6 seems to be an attractive target for therapy to cure trastuzumab-resistant breast cancers. One of the mechanism by which resistance against trastuzumab is induced by IL-6 is through the induction of EMT^22^. We also confirmed this phenomenon as Tr-R cells had higher levels of snail, a biomarker for EMT. The major objective of our study was to study the effects of anesthetic propofol against Tr-R cells and since IL-6 and EMT are mechanistically involved in the process, we found an inhibitory effect of propofol against both IL-6 as well as snail expression. Further, EMT and cancer stem cells are closely related and we not only found increased generation of mammospheres by Tr-r cells, but also their marked inhibition by propofol, which is in agreement with a previously published report^23^. Ironically, propofol can have both pro- as well as anti-tumor effects^3^, but our study supports a action of propofol against Tr-r cells, which is supportive of an anti-tumor effect.

Trastuzumab resistance can have epigenetic origin^18^. We, therefore focused on epigenetic basis of trastuzumab resistance, particularly on the epigenetic basis of IL-6 release. Bioinformatic analysis confirmed miR-149-5p and miR-760 as the two top miRNAs that regulate IL-6 expression. Interestingly, propofol increased the expression of these two tumor suppressor miRNAs, which could then inhibit their target IL-6 and thus sensitize trastuzumab resistance. These findings support an important epigenetic activity of propofol within the tumor microenvironment that can profoundly affect the drug resistance and overall outcome. While our study is the first to describe affect of propofol on miRNA leading to impact on cancer drug resistance, there are reports on affects of propofol on miRNAs but in the context of different outcomes. In particular, similar to our findings regarding the up-regulation of tumor suppressor miRNAs, miR-149-5p and miR-760, propofol has been reported to upregulate other tumor suppressor miRNAs, such as, miR-133a^24^, miR-199a^25^, miR-328^26^, miR-1284^27^ etc. our study is novel not just in the context of trastuzumab resistance but with regards to up-regulation of tumor suppressor in breast cancer in general.

In view of the anticancer effects of propofol described by us here, it is possible that the use of this anesthetic during the breast cancer resection surgery might be beneficial. This was tested and the results reported recently^4^. The work comprised of a large cohort of breast cancer patients who had surgery between 2006 and 2010 and then followed-up until 2016. Even after all exclusions, 592 patients receiving desflurane were compared with 296 patients receiving propofol. The major conclusion of the study was the inability to find any survival benefit with the use of either anesthesia. Of note, there was a small difference in the distant metastases as the patients receiving propofol had fewer (6%) distant metastases, compared to patients receiving desflurane (8%). These numbers are quite close to those reported in another work^28^. Even though the numbers in Huang study^4^ did not receive statistical significance, the trend seems to suggest a possible protecting role of propofol against distant metastases. This also supports our own observations regarding the effect of propofol on lung metastasis in an experimental metastasis model. It is possible that the follow-up in Huang study^4^ was not adequate and an even longer follow-up time would have generated statistically significant results. Such studies are difficult to control for and subtle changes in the patients TNM stage etc. can influence the outcome. A few other reports^29,30^ support the contention that propofol use might not alter the cancer outcome. Even a randomized controlled trial involving 13 hospitals across multiple countries in Argentina, Austria, China, Germany, Ireland, New Zealand, Singapore and the USA, that enrolled patients from 2007 through 2018 reached the same conclusion regarding no particular advantage of using one anesthetic procedure over another^7^.

Supporting a protective role of propofol is an earlier study by Lee *et al*.^6^ which evaluated overall survival in 173 patients who received propofol vs. 152 patients who received sevoflurane during modified radical mastectomy. In this study, the propofol receiving patients had lower rate of cancer recurrence. Also, a study with colon cancer patients^5^ that compared propofol vs. desflurane, similar to breast cancer study above^4^, reported a clear benefit of using propofol as the overall survival was clearly better in patients in propofol group. Thus, the benefits of propofol might not be confined to breast cancer, but might be even enhanced in patients with other cancers. Interestingly, intravenous anesthetics such as propofol might be better than the inhaled anesthetics as the pulmonary complications have been reported to be higher in inhalation based anesthesia, compared to intravenously administered anesthesia^31^. This systemic review further supports our results from the experimental pulmonary metastasis assay.

In summary, we provide first evidence supporting a possible use of anesthetic propofol against resistant breast cancer cells. Our *in vitro* as well as *in vivo* results are supportive of the hypothesis that propofol can epigenetically sensitize trastuzumab breast cancers. Future clinical studies are needed to further explore and evaluate this idea.
